# Modeling the Characteristic Residues of Chlorophyll *f* Synthase (ChlF) from *Halomicronema hongdechloris* to Determine Its Reaction Mechanism

**DOI:** 10.3390/microorganisms11092305

**Published:** 2023-09-13

**Authors:** Min Chen, Artur Sawicki, Fanyue Wang

**Affiliations:** School of Life and Environmental Sciences, University of Sydney, Sydney, NSW 2006, Australia

**Keywords:** photosystem II, chlorophyll, free radical, cyanobacteria, oxygen evolution center, chlorophyll *f* biosynthesis

## Abstract

Photosystem II (PSII) is a quinone-utilizing photosynthetic system that converts light energy into chemical energy and catalyzes water splitting. PsbA (D1) and PsbD (D2) are the core subunits of the reaction center that provide most of the ligands to redox-active cofactors and exhibit photooxidoreductase activities that convert quinone and water into quinol and dioxygen. The performed analysis explored the putative uncoupled electron transfer pathways surrounding P_680_^+^ induced by far-red light (FRL) based on photosystem II (PSII) complexes containing substituted D1 subunits in *Halomicronema hongdechloris*. Chlorophyll *f*-synthase (ChlF) is a D1 protein paralog. Modeling PSII-ChlF complexes determined several key protein motifs of ChlF. The PSII complexes included a dysfunctional Mn_4_CaO_5_ cluster where ChlF replaced the D1 protein. We propose the mechanism of chlorophyll *f* synthesis from chlorophyll *a* via free radical chemistry in an oxygenated environment created by over-excited pheophytin *a* and an inactive water splitting reaction owing to an uncoupled Mn_4_CaO_5_ cluster in PSII-ChlF complexes. The role of ChlF in the formation of an inactive PSII reaction center is under debate, and putative mechanisms of chlorophyll *f* biosynthesis are discussed.

## 1. Introduction

Photosynthesis is a fundamental process that converts light energy into chemical energy. Oxygenic phototrophs use two photosystems (PSI and PSII) that constitute the Z-scheme and the associated generation of assimilatory power. Photosystems are multisubunit complexes in the thylakoid membrane, and PSII absorbs and transforms light energy into chemical energy, resulting in water splitting and oxygen evolution. PsbA (D1) and PsbD (D2) are the core subunits of PSII that exhibit photooxidoreductase activities and convert plastoquinone and water into plastoquinol (QH_2_) and dioxygen (O_2_). The Mn_4_CaO_5_ cluster is the oxygen evolution center of PSII and is directly coordinated with multiple conserved residues of D1 and PsbC (CP43)-glutamic acid-354 (E354), further stabilized through second coordination spheres supported by conserved residues of D1, D2, and CP43 [1,2,3]. The tyrosine-161 (Y161) and histidine-190 (H190) residues of D1 work in tandem to transfer electrons to the reaction center Chl *a* (P_680_), wherein Y161 transfers electrons and H190 acts as a proton acceptor [4,5]. Charge separation involves the transfer of an electron from P_680_, which then forms P_680_^+^, to the primary acceptor (plastoquinone, Q) via an intermediary acceptor, pheophytin (Pheo *a*). 

Amino acid residues surrounding the Mn_4_CaO_5_ cluster affect the PSII oxygen evolving capacity [6,7]. For example, a N181A/S mutation of the D1 protein results in slower O-O bond formation during the water-splitting reaction in *Synechocystis* sp. PCC 6803 [8]. A D170A/S substitution of D1 poisons oxygen evolution capacity, while a D170E mutation is complementary with little effect on activity [9]. Additionally, the substitutions N87A/S, F117L, D170AES, and E189D in D1 result in impaired water-splitting activity and possibly reduced PSII stability [5]. 

Cyanobacteria typically possess multiple *psbA* gene copies with different expression levels that mostly reflect varying environmental conditions [1,10,11,12]. For example, *Synechocystis* sp. PCC 6803 has three *psbA* genes, with *psbA1* upregulated in high light, *psbA2* constitutively expressed [13], and *psbA3* expressed during low oxygen “microaerophilic” conditions. A general name of *psbA′* is given to “microaerophilic” *psbA* genes since other cyanobacteria have a comparable strategy [14]. 

Dark expression of sentinel *psbA* (belonging to the “rogue” D1 group) blocked oxygen-evolving PSII activity through an uncleavable C-terminus and altered amino acid residues surrounding the Mn_4_CaO_5_ cluster in the nitrogen-fixing cyanobacterium *Cyanothece* [1,15,16]. Recently, a D1 variant was renamed “chlorophyll *f* synthase (ChlF)”. It is a far-red light (FRL)-induced paralog of D1 in Chl *f*-producing cyanobacteria [17]. The ChlF-encoding gene participates in the far-red light-induced photoacclimation (FaRLiP) gene cluster, and it is only transcribed under FRL conditions. It is unknown how ChlF catalyzes the formation of chlorophyll *f* (Chl *f*) from Chl *a*, although molecular oxygen is required [18]. The ChlF-encoding gene from *Chroococcidiopsis thermalis* PCC 7203 (AFY86562) was used to replace the D1 subunit-encoding gene in the PSII complex of *Synechocystis* sp. PCC 6803 that resulted in up to ~0.1% Chl *f* of total chlorophyll when the transformant was grown photoheterotrophically with 5 mM glucose at a light intensity of 10–30 μmol photons m^−2^ s^−1^ [19]. ChlF is a D1 paralog that lacks many of the conserved amino acid residues surrounding the Mn_4_CaO_5_ cluster [1]. Those conserved amino acids in ChlF may play important roles in controlling the assembly of an unconventional oxygen-evolving center if ChlF substitutes for D1 in PSII complexes. 

Chl *f* has a 2-formyl in the C2-position of Chl *a* responsible for bulk red-shifted absorption and possesses the most red-shifted absorption in chlorophylls [20]. Chl *f* enables cyanobacteria to utilize habitats in low white light (WL) and high FRL conditions [21,22,23,24,25,26]. Far-red light acclimation is also reported in endolithic cyanobacteria [22,27,28]. Chl *f* is present in PSI and PSII when ChlF is heterologously expressed in *Synechocystis* sp. PCC 6803 and *Synechococcus* sp. PCC 7002 [19,29]. Chl *f* is detected in isolated PSI and PSII systems induced by FRL conditions in the following Chl *f*-producing cyanobacteria: *Chroococcidiopsis thermalis*, *Synechococcus* sp. PCC 7335, and *Halomicronema hongdechloris* [19,29,30,31,32], although quite a few other cyanobacteria have been reported to produce Chl *f* [22,23,24,25,26]. Absorption spectra of naturally occurring Chl *f*-containing photosystems are more red-shifted than those of heterologously produced Chl *f*-containing PS complexes, owing to a relatively higher Chl *f* content [29,32].

There are five *psbA* genes in the *H. hongdechloris* genome. In particular, *psbA2* (*XM38_018880*) codes for WL-D1 (or typical D1) protein in *H. hongdechloris* in WL conditions [33]. In turn, *psbA3* (*XM38_020870*) and *psbA4* (*XM38_037330*) are expressed in WL conditions but at a lower level than *psbA2* [33]. *H. hongdechloris* has paralogous genes encoding FRL-D2 (*XM38_010820*), FRL-CP47 (*XM38_010800*), FRL-CP43 (*XM38_010810*), FRL-PsbH (*XM38_010780*), FRL-PsbO (*XM38_010880*), and FRL-PsbV (*XM38_010890*) subunits of PSII in FRL-grown cells. In addition, FRL-D1 (*XM38_010770*) and ChlF (*XM38_010900*) encoding genes in the FaRLiP gene cluster are upregulated in FRL conditions [33]. 

The Mn_4_CaO_5_ cluster is the center of the water-splitting catalytic region and is directly coordinated with D1 and CP43 in PSII [34]. Cells grown in FRL conditions have a lower O_2_-evolving rate than those grown under WL conditions [35]. ChlF from *H. hongdechloris* has over 70% conserved residues compared with typical D1 proteins. This supports the idea that ChlF may replace the WL-D1 subunit and exhibit similar stability in the PSII complexes [33]. A specific Q127 and D128 substitution in ChlF (the QD motif) is critical for activity and might be involved in binding the newly synthesized Chl *f* [19]. However, it is unclear whether these amino acid residues are directly involved in Chl *f* biosynthesis. 

This report used published data on cyanobacterial PSII structure to characterize the functional motifs of ChlF from *H. hongdechloris* at the primary and 3D-structural modeling levels. The new insights into the functional mechanisms of ChlF and Chl *f* biosynthesis should allow for a better understanding of the various functions of D1 paralogs. 

## 2. Materials and Methods

### 2.1. D1 Protein Sequence Alignment

The FaRLiP gene cluster is used as a biomarker of Chl *f*-producing cyanobacteria. Eighteen cyanobacterial genomes possessing the FaRLiP cluster were selected and obtained from GenBank (NCBI) and the Integrated Microbial Genomes of the DOE joint Genome Institute (Table S1). All putative ChlF and D1 sequences from 18 selected cyanobacterial genomes were retrieved, with a total of 100 D1 paralogs. Identical copies were removed, and 83 sequences (Table S1) were used for sequence comparison. Sequence alignment was generated using Muscle in MEGA 11 and manually refined by predicting the PDB files from each D1 protein group using the SWISS-MODEL online service from ExPasy. The *H. hongdechloris* D2 protein was used as an outgroup. 

### 2.2. The D1 Sequence Comparison

The D1 sequences were retrieved from cyanobacteria containing the FaRLiP gene clusters as described in Wang et al. 2022 [36] and then aligned based on the published D1 tree [11,12]. The subset of D1 homologs (including ChlF sequences) from 18 selected species of cyanobacteria containing the FaRLiP gene cluster were re-aligned with the typical D1 consensus sequence and refined manually according to conserved motifs and predicted secondary structures. The grouped D1 sequences were verified using neighbor-joining (NJ) phylogenetic analysis. The NJ distance tree was determined after 1000 replications using the JTT (Jones, Taylor, and Thornton) model after the deletion of gaps [37]. 

### 2.3. Structure Modeling and Comparison

Three-dimensional structural modeling was performed using Chimera version 1.14 [38], https://www.cgl.ucsf.edu/chimera/download.html (accessed on 1 March 2020). The PSII structural data from *Thermosynechococcus vestitus* BP-1 (also named *Thermostichus vulcanus*) (PDB 5TIS and PDB 3WU2) (non-Chl *f*-producing cyanobacteria) were used as templates to simulate structural models of D1 paralogs from *H. hongdechloris*. The following Uniprot data were predicted: A0A1Z3HIN9, *H. hongdechloris* ChlF (*XM38_010900*); A0A1Z3HJ62, *H. hongdechloris* FRL-D1 (*XM38_010770*); A0A1Z3HKW5, *H. hongdechloris* WL-D1 (*XM38_018880*); and P51765, *T. vulcanus* D1. Note that Uniprot P51765 has a R279P substitution compared with Uniprot P0A444 (D1 of *T. vulcanus*). All stimulated structure models were minimized and optimized after adding H-bonds and the protonation states of conserved charged amino acid residues, such as histidine (H) and glutamic acid (E). All predicted models were refined using the Swissprot database (https://swissmodel.expasy.org, accessed on 1 March 2020) against the template file (5TIS.pdb). SWISS-MODEL allows the production of a 3D model of heteromeric complexes [39], such as FRL-PSII complexes stimulated using PSII subunits from the FaRLiP cluster (FRL-PSII subunits). The individual 3D models of ChlF, FRL-D1, and WL-D1 of *H. hongdechloris* were used in place of D1 from *T. vulcanus* (PDB 5TIS). The heterodimers of D1/CP43/CP47/D2 using WL-PSII and FRL-PSII subunits from *H. hongdechloris* were predicted against the PDB 5TIS template. 

Hydrogen atoms and hydrogen bonds are not observable in most crystal structures. However, many cofactors in PSII, such as Pheo *a* and the plastoquinones Q_A_ and Q_B_, interact with the D1 protein via hydrogen bonds (H-bonds). Therefore, hydrogen bonding is important to determine the physical structure parameters. The following criteria are needed to form H-bonds in a predicted protein structure: (1) donor atom−Acceptor atom <3.9 Å; (2) Hydrogen−Acceptor atom <2.5 Å; (3) donor atom−Hydrogen atom−Acceptor atom >90.0°; (4) adjacent atom to acceptor A′−Acceptor atom−Donor atom >90.0°; and (5) adjacent atom to acceptor A′−Acceptor atom−Hydrogen atom >90.0° as discussed in Torshin et al. [40] and McDonald and Thornton [41]. H-bond analysis was predicted and verified using the tools in Chimera. 

## 3. Results

### 3.1. Alignment and Phylogenetic Relationship of D1 Paralogs in Chl f-Producing Cyanobacteria

There are 19 published genomes containing the FaRLiP gene cluster. Eighteen of them are complete or incomplete whole genomes, and most are labeled as ‘permanent drafts’ (Table S1). D1-encoding genes and their paralogs were retrieved from GenBank (NCBI) and the Integrated Microbial Genomes of the DOE joint Genome Institute and verified after BlastP comparison and annotation. 

**Figure 1:**
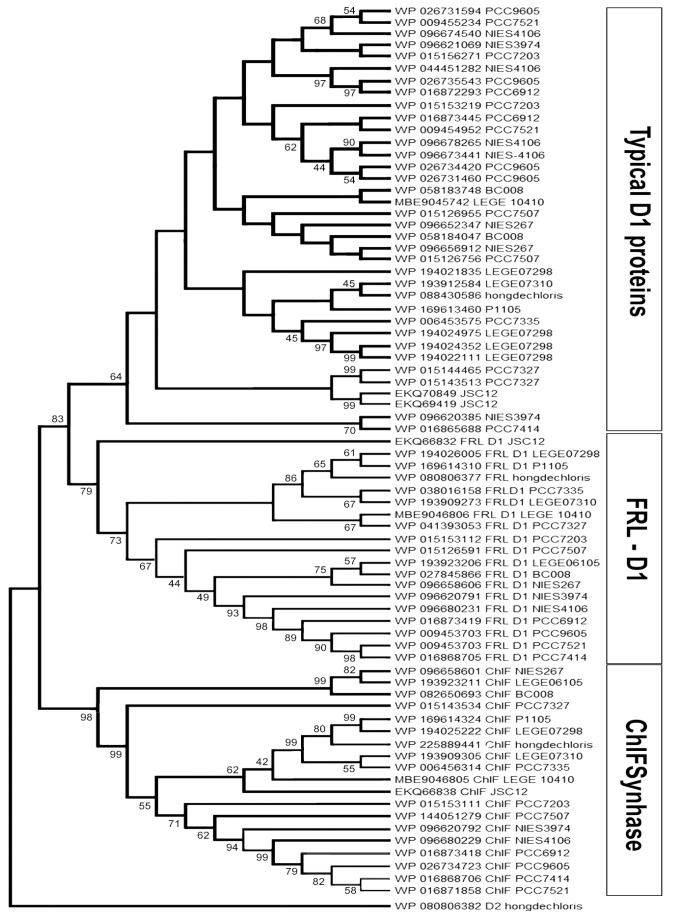
The neighbor-joining distance tree of D1 paralogs in Chl *f*-producing cyanobacteria. The distance tree was generated using the neighbor-joining (NJ) method in MEGA 11. *H. hongdechloris* D2 protein (WP080806382) was used as an outgroup. All sequence IDs and strain numbers used for the names and the sequence accession are listed in Table S1. The branch support numbers were calculated by 1000 replications of NJ and were presented if the value was above 40%. Typical D1 groups include the dominant D1 protein (WL-D1) of *H. hongdechloris* grown under white light conditions; FRL-D1 (far-red light D1), a group covering genes encoding D1 proteins in the FaRLiP cluster; and ChlF synthase, a new group of D1 paralogs that synthesize chlorophyll *f*.

**Figure 2:**
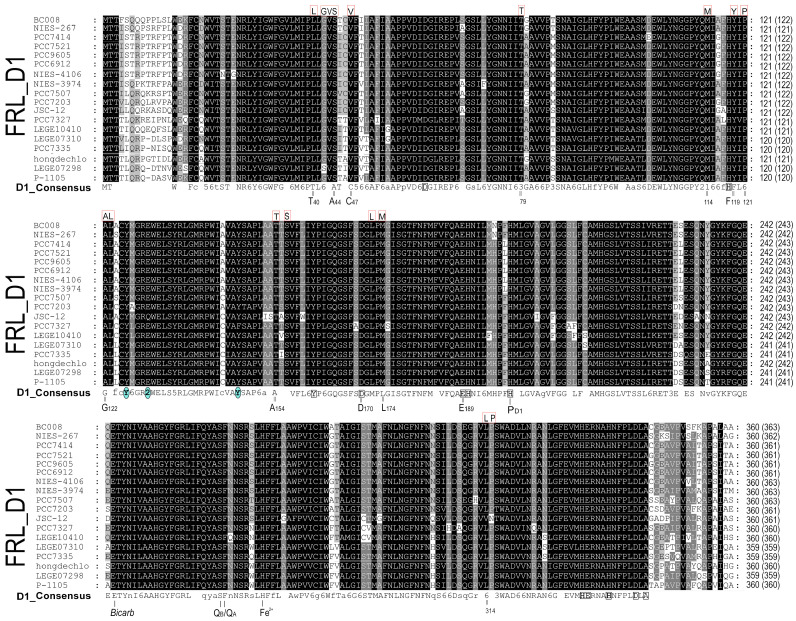
Far red light (FRL)-D1 protein sequence alignment supported by phylogenetic analysis in Figure 1. The specific conserved sites of FRL-D1 are highlighted by red-framed boxes. The aligned sequence lengths are labeled as ‘numbers (numbers)’ at the end of each sequence. The numbers outside parentheses are aligned sequence lengths matching the D1_consensus (unaligned gaps were removed), which is the number used in the models. The numbers inside parentheses represent the original length. The D1_consensus sequence surrounding the Mn_4_CaO_5_ cluster is highlighted in square boxes and bold fonts. The residues interacting with Pheo *a* in the D1_consensus sequence are highlighted with blue-colored circles. The D1_consensus sequence was generated by Genedoc based on 50 typical D1 protein sequences. The conserved residues from 18 FRL-D1 sequences are highlighted with red-square boxes on top of the alignments, and corresponding amino acid residues are numbered under the D1_consensus sequence. The numbers in the D1_consensus represent the conservative substitutions: 2 = Q/E; 3 = S/T; 5 = MFY; 6 = F/I/L/M/V.

The D1 homologs can be divided into three main groups: WL-D1 (Typical D1), FRL-D1, and FRL-ChlF (Figure 1). According to protein alignments, FRL-D1 proteins possess all essential conserved motifs of the D1 Protein and contribute to functional Chl *a*/*f*-binding PSII under FRL conditions, including the high-affinity Mn-binding sites of D170 and E189 (Figure 2). The similar structures and conserved motifs among 18 FRL-D1 sequences can be defined as L40, G42, V43, S44, V47, T79, M114, Y119, P121, A122, L123, T154, S156, L172, M174, L314, and P315 (Figure 2).

**Figure 3:**
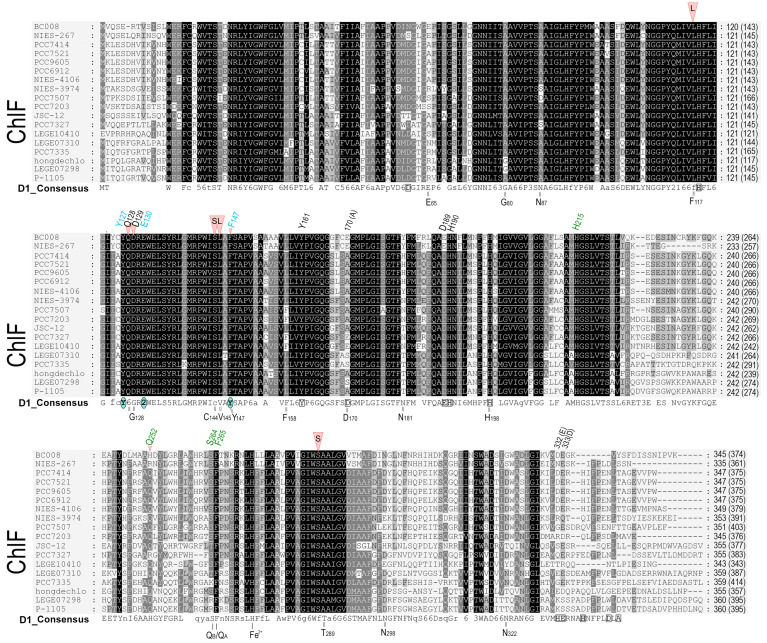
ChlF protein sequence alignment supported by phylogenetic analysis in Figure 1. The specific conserved sites of ChlF are highlighted by red triangles. The aligned sequence lengths are labeled as ‘numbers (numbers)’ at the end of each sequence. The numbers outside parentheses are aligned sequence length matches with D1_consensus (removed unaligned gaps), which is the number used in the models. The numbers inside parentheses represent the original length. The D1_consensus sequence surrounding the Mn_4_CaO_5_ cluster is highlighted with square boxes and bold fonts labeled in the D1_consensus sequences. The noticeable changes in amino acid residues at the positions associated with the Mn_4_CaO_5_ cluster are labeled on the top of aligned ChlF sequences (black-colored fonts). The residues interacting with Pheo *a* in the D1_consensus sequence are highlighted by blue-colored circles, and their corresponding sites are labeled on top of the aligned ChlF sequences in blue-colored fonts. The amino acid residues surrounding the Q_B_ site (as presented in Section 3.4 below) are labeled on top of the aligned ChlF sequence, indicated as green-colored fonts. The D1_consensus sequences were generated by Genedoc based on 50 typical D1 protein sequences. The specific amino acid residues of ChlF are marked in a pink triangle on top of the alignment, and the corresponding amino acid residues are numbered under the D1_consensus sequence. The numbers in the D1_consensus represent the conservative substitutions: 2 = Q/E; 3 = S/T; 5 = MFY; 6 = F/I/L/M/V.

ChlF forms a distinct branch from FRL-D1 and other D1 groups (Figure 1). The similar residues among ChlF synthase sequences can be characterized as: L117, Q127, D128, S144, L145, F147, Q 252, and S285 (Figure 3), including the previously reported QD motif at sites 127 and 128 (Q152 and D153 of ChlF from *H. hongdechloris*) [19]. The sequence comparison confirmed that the H252Q substitution is dominant in ChlF sequences, and only two cases of H252R substitutions were observed in *Pleurocapsa* sp. PCC 7327 and *Calothrix parasitica* NIES-267 (Figure 3). Interestingly, there is almost no overlap between the specific FRL-D1 motifs and ChlF sequences (Figure 2 and Figure 3). ChlF demonstrated significant changes in the conserved residues surrounding the Mn_4_CaO_5_ cluster, although the most significant changes are not consistent in ChlF sequences, as highlighted in Figure 3. For example, the conserved residue D170 in the typical D1 sequence is A, S, and E in ChlF (Figure 3). Campbell et al. reported that Mn^2+^ was oxidized and stabilized as Mn^4+^ in a D170E mutant, resulting in a photoactivating PSII that produces oxidizing manganese (Mn^4+^) but without O_2_ evolution [42,43]. The substitutions N87A/S and D170AES are noticed in ChlF alignment, and those changes in D1 impair oxygen evolution activities [5]. Thus, the replacement of D1 with the ChlF paralog may cause the loss of oxygen evolution. 

### 3.2. The Modeling of D1 Paralogs from H. hongdechloris

ChlF shares over 70% of its residues with D1 proteins. This allows for the generation of a reliable 3D model using structural templates of known PSII structures in cyanobacteria. PDB 3WU2 from *T. vulcanus* was initially used as the template for modeling ChlF. ChlF interaction with other PSII subunits pertain to FRL-regulated structural reassembling subunits, especially FRL-D2 and FRL-CP43. The initial modeling trials without added H-atoms generated poor individual models of ChlF, FRL-D1, and WL-D1 (typical D1) since the estimated overlap (3.5 Å) scores were well below 0.7 (Table S2). This score refers to the proportion of Cα atoms within 3.5 Å of the proposed modeling structures. The modeling of *H. hongdechloris* WL-D1 was relatively well matched against 3WU2-D1, including the positions of all functional motifs. Further, FRL-CP43 modeling was improved after adding H-atoms to the initial model (Table S2). The modeling results were further improved by excluding a certain number of amino acids at the N- or C-termini that do not align with the structural template. The estimated overlap scores were significantly improved to well above 0.7 after using the truncated sequences. The estimated overlap (3.5 Å) score of the truncated ChlF models was similar and above 0.9, with model three showing the highest score of 0.994 (Table S2).

### 3.3. Pheophytin a Binding Environment

**Figure 4:**
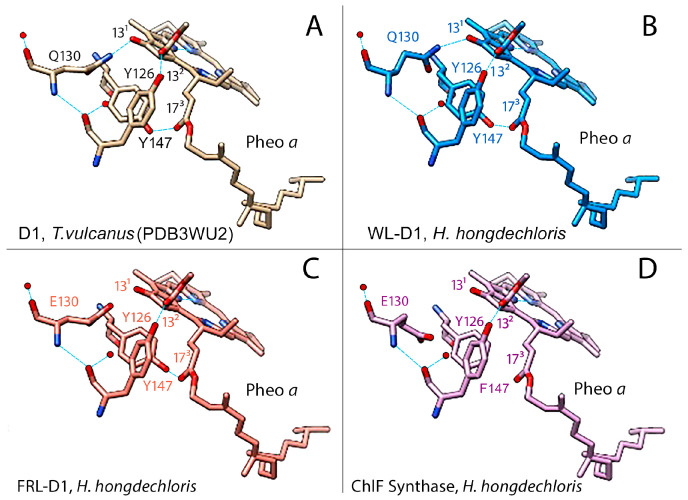
Modeling of the pheophytin *a* (Pheo *a*) binding region in WL-D1, FRL-D1, and ChlF of *H. hongdechloris. T. vulcanus* (PDB 3WU2) was used as a template. The amino acid positions known to form H-bonds (light blue lines) with Pheo *a*: Y126 (Tyr-O•••H•••O=C13^2^-Pheo *a*); Q130/E130 (N-H or O-H) (Q-N•••H•••O=C13^1^-Pheo *a*) or (E-O•••H•••O=C13^1^-Pheo *a*), and Y147 (Tyr-O•••H•••O=C17^3^-pheo *a*) are included to compare with the predicted H-bonds. (**A**) The crystal structure of D1 of *T. vulcanus* (PDB 3WU2) was used as a template for the modeled structure. (**B**–**D**) represent models of the Pheo *a* binding regions of WL-D1, FRL-D1, and ChlF synthase, respectively. Dark blue rods, nitrogen atoms; red rods, oxygen atoms; and dotted light blue lines, H-bonds. Note: FRL-D1 and ChlF synthase have E130, which may have H-bonds with the 13^1^-keto of Pheo a, and these bonds might be stronger than Q130 (Table S3).

Both FRL-D1 and ChlF have conserved E130 sites (Figure 2 and Figure 3). The strength of the hydrogen bond between this residue and Pheo *a* could be changed. The structures were further modeled by SWISS-MODEL against the PDB 5TIS template from *T. vulcanus* [44]. The FRL-D1 model retained H-bonds with Pheo *a* and had a similar distance to the PDB 5TIS template, although the substitution of E130 (protonated at OE2) provided a stronger (shorter) H-bond than Q130 in the template (Figure 4). Hence, FRL-D1 demonstrated a comparable Pheo *a* binding profile to that in highlight acclimated cyanobacteria or plant PSII systems [45,46]. 

ChlF modeling following the template placement of ligands generated H-bonds when E130 was selectively protonated at OE2; however, H-bonds were absent in non-protonated E130 and protonated E130-OE1 situations (Figure 4). ChlF retains the Y126 H-bond with C13^2^ of Pheo *a* to provide a comparable H-bond distance to the template. However, the Y147F substitution in ChlF results in a loss of the H-bond, Y147-O•••H•••O2-C17^3^-Pheo *a* (Figure 4 and Figure S1, and Table S3). This contributes to the lessened interaction with Pheo *a*, although the impact of the missing H-bond with C17^3^ Pheo *a* is unknown. The potential change in orientation of Pheo *a* due to the loss of an H-bond at C17^3^ Pheo *a* enhanced the strength of the H-bond with E130 in ChlF (Figure S1). Using flourescence relaxation measurement, Rappaport et al. observed acceleration of the S_2_Q_A_^−^ recombination process in the Q130E mutant [45]. Thus, E130 in FRL-D1 and ChlF could decrease the recombination rate of ^1^[P_680_^+^/Pheo *a*^−^] to P*_680_ in order to acclimate to changed light environments. 

### 3.4. Q_B_ Binding Environment

**Figure 5:**
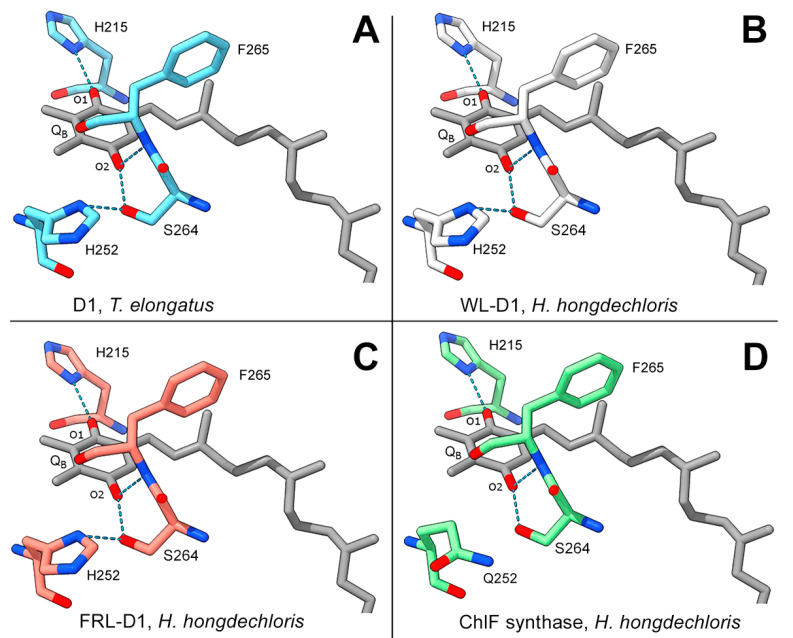
Modeled D1 paralogs surrounding Q_B_. Modeling of WL-D1, FRL-D1, and ChlF of *H. hongdechloris* against the PDB 5TIS template from *T. vulcanus* (Young et al. 2016 [44]) was performed using SWISS-MODEL. The conserved H-bonds of *T. vulcanus* D1 are shown as a positive control (**A**) to indicate modeling validity. (**B**) *H. hongdechloris* WL-D1; (**C**) *H. hongdechloris* FRL-D1; and (**D**) *H. hongdechloris* ChlF synthase. Q_B_ molecular bonds are in gray; residues from the *T. vulcanus* D1 template are in light blue; residues from *H. hongdechloris* WL-D1 are in white; residues from *H. hongdechloris* FRL-D1 are in orange; and residues from *H. hongdechloris* ChlF are in green; dark blue rods, nitrogen atoms; red rods, oxygen atoms; dotted light blue lines, H-bonds.

The typical D1 subunit has three H-bonds with two keto groups of Q_B_: H215-N-H•••O=keto Q_B_^−^O1, S264-O-H•••O=keto Q_B_^−^O2, and F265N-H•••O=keto Q_B_^−^O2. Additionally, S264 forms an H-bond with H252, H252-N-H•••O-S264 (Figure 5). Modeling of FRL-D1 and WL-D1 of *H. hongdechloris* simulated the H-bonds of H252-N-H•••O-S264 and the Q_B_ coupling environments were potentially unchanged (Figure 5). H252 protonation supports the protonation of Q_B_ to form Q_B_H_2_, an important electron transfer towards oxygen-evolving activities [47]. However, ChlF has a H252Q substitution that is known to affect electron transfer (Figure 5D). The lack of an H-bond between Q252 and S264 inhibits the putative protonation of Q_B_ and conversion of Q_B_H_2_ [2], and mutant H252Q exhibitsed rapid electron transfer from Q_A_^−^ to Q_B_ [48]. The common substitution of H252Q (Figure 5D) implies that there is a more flexible structure in this region because H-bond formation should precede protonation to preserve some of the H-bonds supporting Q_B_.

### 3.5. Putative Chl f-Binding Region of ChlF-PSII Complexes

**Figure 6:**
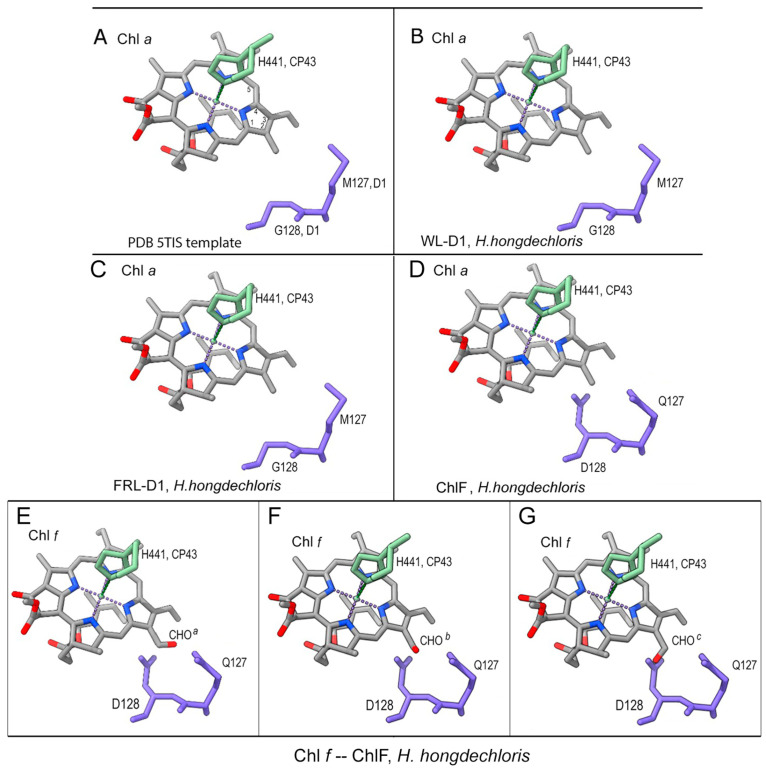
Position of the QD motif of ChlF compared with conserved MG residues from other D1 proteins. (**A**) Template 5TIS; (**B**) modeled *H. hongdechloris* WL-D1; (**C**) modeled *H. hongdechloris* FRL-D1; (**D**) modeled *H. hongdechloris* ChlF; and (**E**–**G**) Chl *a* was replaced with Chl *f* at the position of the modeled ChlF. The C2-CHO group of Chl *f* was shown at different orientations. (**E**), position a; (**F**), position b; and (**G**), position c, of the oxygen atom of CHO. Chlorophyll molecular bonds are gray; residues 127 and 128 of D1 and D1 paralogs are purple-blue. The histidine (H441) of CP43 is in green. Dark blue rods, nitrogen atoms; red rods, oxygen atoms; dotted lines, H-bonds.

The specific QD motif in ChlF probably plays a biochemical role in the synthesis and binding of Chl *f*, which is at the position of CP43 binding to Chl *a* through the axial ligand, H441 [19]. ChlF was modeled with multiple rotamers to test the acceptable placement of Q127 or/and D128 substitutions. There were clashes with the original placement of D128 and the Chl *a*/Chl *f* atoms. Position-c of the three putative spatial orientations of the formyl group of Chl *f* (designated C2-CHO^a/b/c^) showed obvious clashes, while positions-a and -b only showed clashes with the C2 and C3 sub-groups of Chl *a* (Figure 6). Potential H-bonds were formed with D128-OE2 of ChlF after manually placing Chl *f* in the position of Chl *a* and confirmed after minimization and refinement of the model (Figure S2). However, no H-bonds were formed between the C2-formyl group of Chl *f* and protonated D128 at OE1 or when D128 was unprotonated (Figure S2). This was further supported by the minimized modeling structure of the ChlF-PSII complex. Therefore, protonated D128 at the OE2 position is the optimized situation in the modeled ChlF structure.

Further minimization steps included: (1) 2-formyl modification of Chl *a*-H441 of CP43 (CLA506.C); (2) H-bond formation; (3) protonation and H-atom addition of E130 OE2 and D128 OE2; (4) movement of Chl *f* into a favorable position for H-bond formation with D128 OE2; and (5) the formation of a H-bond between D128 OE2 and Chl *f*. The refined and minimized structure proposed that the ideal placement occurred in rotamer model seven if Chl *f* replaced Chl *a* in the region (Figure S2). 

### 3.6. Mn_4_CaO_5_ Cluster Modeling

There is no consensus sequence for the Mn_4_CaO_5_ cluster following the alignment of ChlF sequences, despite some organisms retaining some of the typical amino acids (Figure 3). The significant changes in ChlF at the sites coordinating the Mn_4_CaO_5_ cluster (Figure 3) suggest that water splitting in a PSII-ChlF complex is impossible owing to the uncoupled Mn_4_CaO_5_ cluster. Multiple subunits of the *H. hongdechloris* PSII complexes were replaced with subunits of FRL-D1/D2/CP43/CP47 and/or ChlF/D2/CP43/CP47 and modeled using SWISS-MODEL. 

**Figure 7:**
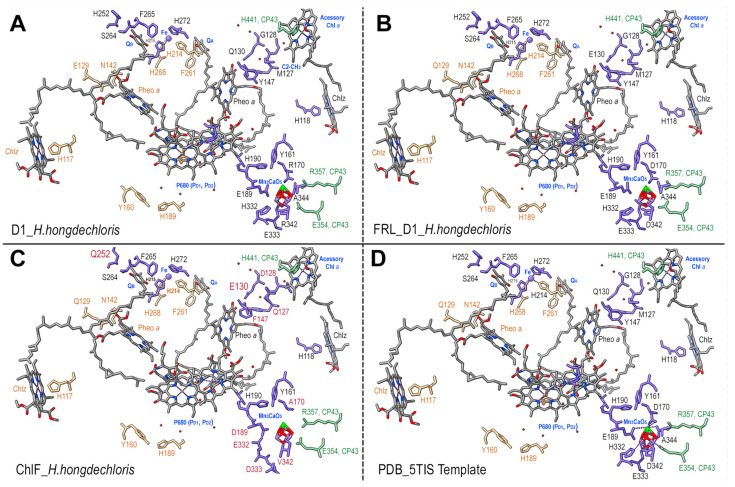
Models of PSII ligands involved in electron transport and water splitting. (**A**) Typical PSII D1 of *H. hongdechloris* (WP088430586); (**B**) *H. hongdechloris* FRL_D1 (WP080806377); (**C**) *H. hongdechloris* ChlF (WP 225889441); and (**D**) the PDB 5TIS template. The amino acids from CP43 are in green. The cofactors are in gray except for the Mn_4_CaO_5_ cluster. The unique ChlF residues are labeled in red. The D1 residues are in black fonts; the D2 residues are in brown fonts; and the partial amino acid side-group bonds are in brown colors. Red rods, oxygen atoms (which also represent Mn in the Mn_4_CaO_5_ cluster); dotted lines, H-bonds; and dark green, Ca in the Mn_4_CaO_5_ cluster.

**Figure 8:**
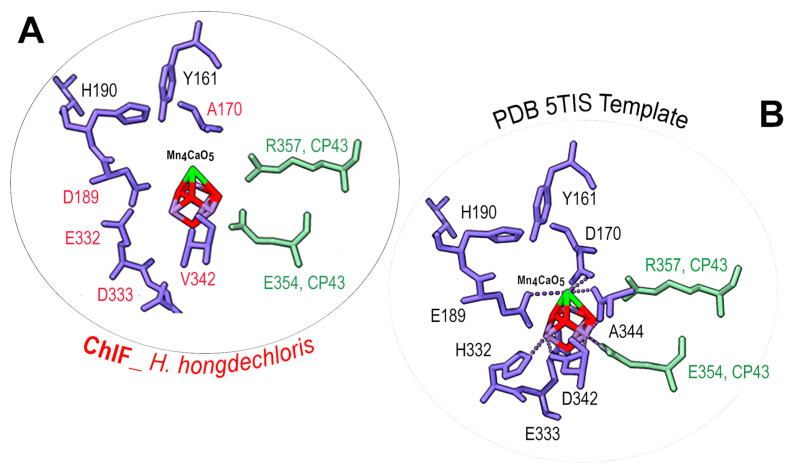
Detailed amino acid ligands surrounding the Mn_4_CaO_5_ cluster. (**A**) A detailed model of ChlF from *H. hongdechloris* (WP 225889441) and (**B**) structural details of the PDB 5TIS template. The conserved (black fonts) and different (red fonts) amino acids surrounding the Mn_4_CaO_5_ cluster. ChlF residues are in purple-blue. CP43 amino acids are in green. The red rods represnt the Mn in the Mn_4_CaO_5_ cluster; the green rod is the Ca in the Mn_4_CaO_5_ cluster; and the dotted lines are H-bonds.

The detailed environment surrounding the Mn_4_CaO_5_ cluster was demonstrated in Figure 7. The PSII-ChlF model showed conserved axial ligands H118 (D1), H198 (D1), H117 (D2), and H197 (D2), together with H-bonds with Pheo *a*, Q_B_, and the conserved Y161/H190 pair. Additionally, the presence of two conserved amino acid residues, D/E189 (Mn1, Ca) of ChlF and E354 of CP43 (Mn2 and Mn3), suggests an Mn_4_CaO_5_ cluster may still be able to assemble or be present in the PSII-ChlF complex in an unconventional way (Figure 8). E189 provides a ligand to Mn in the metal cluster, but D189 from ChlF in *H. hongdechloris* showed no H-bond ligand to the metal cluster (Figure 8). D189 represents the conserved site in 16 ChlF sequences, except ChlF from *Mastigocoleus testarum* BC008 and *Calothrix parasitica* NIES-267, supporting the dysfunctional oxygen evolution center in PSII-ChlF complexes (Figure 3). The D1 mutant of E189D was reported to abolish water oxidation [4]. 

Additionally, D170 involves the light-driven one electron oxidation of Mn^2+^ to Mn^3+^, and it is A170 from *H. hongdechloris* ChlF (Figure 8). The residues H332, E333, and D342 at the C-terminal of the typical D1 sequence are important for assembly of the Mn_4_CaO_5_ cluster, and they are E332, D333, and V342 in the *H. hongdechloris* ChlF sequence (Figure 3). According to the model, the ligands with the Mn_4_CaO_5_ cluster are lacking in the ChlF sequence, indicating that these residue substitutions could lead to an altered function and efficiency of PSII oxygen evolution. The E333D mutant in *Synechocystis* PCC 6803 showed no photosynthetic oxygen evolution, and the H332E mutant appeared to induce rapid charge recombination between Q_A_^−^ and Yz^ox^ [49]. Therefore, the water splitting process is presumed deficient, and ChlF probably has an alternative structure and function due to the several amino acid substitutions that are critical for coordinating the Mn_4_CaO_5_ cluster.

## 4. Discussion

The genes encoding FRL-D1 and ChlF are in the FaRLiP cluster under the same regulatory mechanism [33]. The different functions of D1 paralogs (FRL-D1 and ChlF) support the hypothesis of two different forms of PSII complexes assembled under FRL conditions. One of the substituted FRL-PSII complexes contains FRL-D1 and other FRL-PSII subunits. FRL-D1 proteins possess all essential conserved motifs of the D1 protein and agree well with the published FRL-PSII cryo-EM structure [50], although the alignment of 18 FRL-D1 sequences showed variations at the positions of L124 with A/S/C and L206 with three species having F206 at the position (Figure 2). The presence of the critical residue coupling of the oxygen-evolving Mn_4_CaO_5_ cluster in FRL-D1 suggests a functional Chl *a*/*f*-binding PSII under FRL conditions as PSII-FRL-D1 complexes (Figure 2). Therefore, the FRL-PSII complex conducts PSII-performing charge separation and water-splitting reactions under FRL conditions, which is consistent with the recent structure of PSII complexes acclimated to FRL conditions [50,51,52]. Meanwhile, the FRL-PSII complex (PSII-ChlF) containing the ChlF subunit instead of D1 proteins (FRL-D1 or WL-D1) plays a certain role in Chl *f* biosynthesis. PSII-ChlF contains the previously described QD motif [19] and conserved L117, Q127, D128, S144, L145, F147, Q 252, and S285 in the 18 ChlF sequences. Six out of eight specific amino acid residues surrounding Mn_4_CaO_5_ are not shared with typical D1 and FRL-D1, although some changes are various between ChlF sequences (Figure 8). H332 from typical D1 changed to H332D, H332E, H332N, H332R, H332S, H332Q, and H332K in ChlF (Figure 3), and experimental data indicated most mutants abolished oxygen evolution capability and only mutants of H335S and H335Q retain 10–15% oxygen evolution rate [49]. The PSII-ChlF structural model supports a new function of ChlF, although it shares >70% homology with D1. 

### 4.1. Proposed Uncoupled Mn_4_CaO_5_ Cluster in PSII-ChlF Complexes

The center of the oxygen-evolving complex is the Mn_4_CaO_5_ cluster. It is stabilized with reaction center proteins D1 and D2 and is surrounded by inner antenna proteins CP47 (near D2) and CP43 (near D1) [53]. Water splitting proceeds in a multi-step process whereby the carefully positioned antenna chlorophylls of CP43 and CP47 transfer their excitation energy to the reaction center chlorophylls (P_D1_ and P_D2_) of D1 and D2, respectively. Excited P_680_ donates electrons to primary acceptor Pheo *a* which transmits them to Q_A_ and Q_B_. Oxidized P_680_^+^ is reduced by an electron donated via Y161 (Y_Z_, D1) by the chemical splitting of water into oxygen. This occurs because the Mn_4_CaO_5_ cluster undergoes a series of redox reactions known as Kok’s “oxygen clock” [half-reactions: 2H_2_O → 4H^+^ + 4e^−^ + O_2_, and 2Q_B_ + 4H^+^ + 4e^−^ →2Q_B_H_2_]. Y_Z_ is an electron donor for P_680_^+^ in PSII and an electron carrier from the Mn_4_CaO_5_ cluster to the electron acceptor P_680_^+^.

ChlF synthase is a paralog of the reaction center protein D1; however, structural modeling (Figure 3 and Figure 7) confirmed the differences between ChlF and FRL-D1 and proposed an uncoupled Mn_4_CaO_5_ cluster in the PSII-ChlF complex. The proposed unconventional Mn_4_CaO_5_ cluster is supported by noticeable variations in ChlF sequences in the region of the 241-DEEE motif (Figure 3), the position for coordinating the bicarbonate in the typical D1 [1]. This is consistent with the hypothesis of “super-rogue” D1 proteins [1,11]. Additionally, N298 on the D1 protein is required for oxygen evolution, and mutants of N298D and N298E will impair oxygen evolution activity [54]. The D298 and E298 substitutions occur in 15 ChlF sequences (Figure 3). 

P_680_^+^ is a powerful oxidant that facilitates the extraction of electrons from the Mn_4_CaO_5_ cluster via redox active Y_Z_. Hence, reduction of P_680_^+^ might occur via cytochrome *b*559 and Chl *a*, or via Y_Z_ and Y_D_ in the absence of a coordinated active Mn_4_CaO_5_ cluster. Electron donation to P_680_^+^ via Y_Z_ is still possible with the aid of Y_D_ at a higher pH environment; that is, Y_D_ can outcompete Y_Z_ in the reduction of P_680_^+^ at a higher pH [55]. The altered electron transfer in the PSII-ChlF complex may enable the reduction of P_680_^+^ by the oxygenation of Chl *a* at the C-2 position to produce Chl *f*. 

### 4.2. Changed Electron Transfer Pathways in Dysfunctional PSII-ChlF Complexes

ChlF has an unconventionally coordinated Mn_4_CaO_5_ cluster (Figure 8). According to experimental data, the significant changes surrounding the Mn_4_CaO_5_ cluster could not support water oxidation and oxygen evolution even if they were present in PSII-ChlF complexes [4,5,6,7,49,54]. In addition, the changes at the D1 conserved sites N87, F117, D170, and E189 in ChlF (Figure 3) reduced PSII stability and included impaired water-splitting activities [5]. Another noticed change around Pheo *a* includes the feature substitution Q128D129 motif in ChlF (Figure 7). The conserved site of F147 in ChlF could shift the E_m_ (Pheo *a*/Pheo *a*^−^) to more positive values and decrease the recombination rate of ^1^[P_680_^+^/Pheo *a*] to P*_680_ [56]. However, ChlF-PSII complex has conserved Y_Z_ and Y_D_ together with H-bond partners H190 (D1) and H189 (D2) at similar modeled distances to those of D1. On the whole, this suggests that there is an altered electron transport efficiency from P_680_^+^ to primary and secondary acceptors. The presumed non-functional, uncoupled Mn_4_CaO_5_ cluster in PSII-ChlF complexes suggests that Y_Z_ and Y_D_ supply the electrons required for the completion of redox reactions. Y160 of ChlF is conserved compared to other D1 sequences; however, this residue is not needed for the catalytic formation of Chl *f* [57]. Interestingly, the F147Y substitution in ChlF could cause an undelivered (or slower) electron at the level of Pheo *a*. Additionally, electron transfer within PSII is possible from a photooxidized reaction center to Pheo *a*, Q_A_, and Q_B_. DCMU (3-(3,4-dichlorophenyl)-1,1-dimethylurea) is an inhibitor of the Q_B_ binding site and inhibits Chl *f* synthesis [57]. Therefore, the light-driven redox chemistry of PSII without the coupled Mn_4_CaO_5_ cluster would play an active role in Chl *f* biosynthesis.

PSII electron transfer follows the resonance energy migration within the light-harvesting antenna with the resulting excitation of the reaction center primary donor (chlorophyll dimer P_680_, or P_D1_ and P_D2_) while the primary acceptor (Pheo *a*) reduces the primary quinone (Q_A_). This causes a reduction of oxidized P_680_^+•^ by Y_Z_ of D1 due to water oxidation by the coordinated Mn_4_CaO_5_ cluster. Impairment of the Mn_4_CaO_5_ cluster suggests that there is a longer-lived P_680_^+•^ radical [58], and superoxide or H_2_O_2_ may be released as a long-lifetime free radical [59,60]. The expression of a ChlF-encoding gene in a PSII-less mutant (D1/D2 double mutant) yielded Chl *f*, and its production was regulated by light-driven electron transfer [57]. Hence, a longer-lived P_680_^+•^ radical is required for Chl *f* biosynthesis. 

### 4.3. Reaction Mechanism of Chl f Biosynthesis Pathway

**Figure 9:**
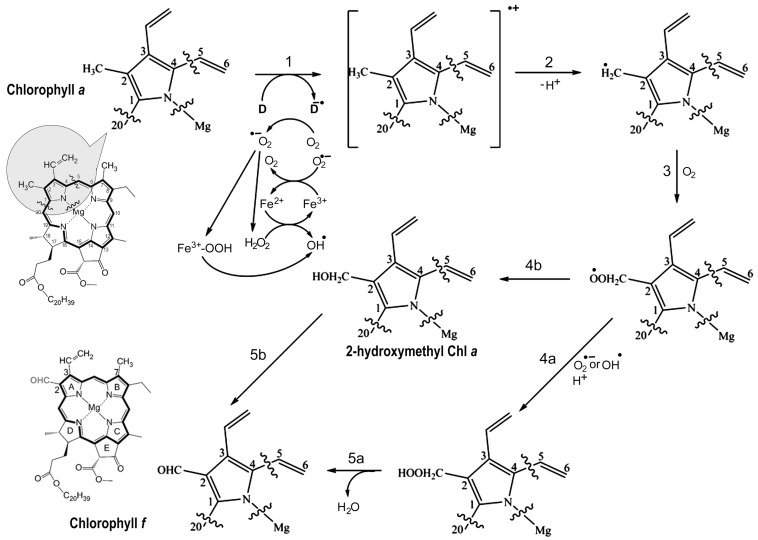
Proposed chlorophyll *f* biosynthesis reaction schemes. The proposed free-radical reaction schemes for chlorophyll *f* synthesis from chlorophyll *a* involve the transformation of a C2 methyl group in Chl *a* into a formyl group in Chl *f*. The first step involves the formation of an anion radical (D^−•^) that might be the overexcited Pheo *a* in an oxygenated aqueous environment or a light-activated radical species through the coupled electron donation by the Chl *a* molecule. Deprotonation forms the radical methyl cation of the Chl *a* substrate (Step 2), followed by reaction with oxygen to generate a peroxo radical (Step 3) that reacts with a superoxide or hydroxy radical (Step 4a) produced through the reduction of oxygen by light-activated reductants, such as Pheo *a* (−610 mV) and Q_A_ (−80 mV). This forms the unstable hydroperoxymethyl Chl, which is further hydrolyzed to the formyl group (Chl *f*). Alternatively, the peroxo radical may decompose into the hydroxymethyl Chl derivative (Step 4b), which further reacts in an unknown pathway to form the formyl group (Step 5b). The partial structure of chlorophylls showed the side group containing the C2-methyl of Chl *a* (shaded region).

Oxidation of a methyl group to a formyl group is a difficult task because CH_3_ is a chemically stable side group. Free radical formation can be specifically involved [61,62,63]. Pheo *a* on the acceptor side of PSII may form a singlet oxygen or hydroxyl free radical. Figure 4 suggests that the specific residues of F147 in ChlF cause a loss of the H-bond at C17^3^-Pheo *a* and a potential slower electron transfer rate, although there is no available experimental data, such as a constructed D1 mutant of Y147F (Figure 4). Non-heme iron may also contribute to the formation of singlet oxygen or hydroxide radicals in the presence of light and aerobic conditions [64,65,66,67]. The inhibition of Chl *f* synthesis by DCMU implied that electron transports by the primary and secondary acceptors Pheo *a*, Q_A_, and Q_B_ were involved in the reaction chemistry [57]. As is known in PSII, the dominant pathway of charge recombination goes through the radical pair of [P_680_^+^/Pheo *a*^−^], and an additional non-radiative pathway could proceed through repopulation of P*_680_ from ^1^[P_680_^+^/Pheo *a*^−^], which was observed in D1 mutant Q130E in *Synechocystis* PCC 6803 [56]. We proposed the reaction scheme of Chl (Chlide) *f* synthesis from Chl (Chlide) *a* via free radical chemistry (Figure 9). Pheo *a* excitation in an oxygenated environment produces an anion radical (D^−•^) via singlet and triplet excited states coupled to electron donation by Chl *a*. The cation-radical of the Chl *a* methyl group is produced via deprotonation. This cation-radical reacted with dioxygen to form a peroxide, which was further oxidized to superoxide or hydroxyl radical (Figure 9, step 4a) via reductants Pheo *a* (−610 mV) or Q_A_^−^ (−80 mV) [68,69].

Peroxide radicals can decompose into hydroxymethyl-Chl derivatives and further react to form the formyl group in an unknown pathway (Figure 9, step 4b). Therefore, the altered electron pathway from P_680_^+^ to Pheo *a*, Q_A_, and Q_B_ in the PSII-ChlF complex drives Chl *f* synthesis. The non-heme iron of PSII might not be involved in this oxygenation in the proposed mechanism; this differs from the biosynthetic mechanism of Chl *b* [70]. 

Chemical synthesis involving the oxidation of aromatic molecules with a methyl sidechain is often accomplished by free-radical chemistry using water as a proton source under elevated temperatures; over-oxidation of the carboxylic acid is prevented by addition of a catalyst such as copper [71]. More recently, a radical-free mechanism has been more specific for the synthesis of formyl groups [72]. ChlF has a likely impaired oxygen-evolving cluster; therefore, there is an increased potential for light-induced photoinhibition (even under low visible light) [73]. Two important photoinhibition sites in PSII include charge separation between P_680_^+^ and Q_A_^−^ and electron transfer from Y_Z_ to P_680_^+^ [74]. Photoinhibition can cause carotenoid and chlorophyll radicals (from photopigments adjacent to P_680_ and accessory Chls) following the loss of Y_Z_ and Y_D_ radicals [75]. Accessory Chls and carotenoids in PSII have been proven to represent sites of photoinhibition damage following a hydroxylamine treatment [76]. It is unclear if ChlF is degraded during photoinhibition since detailed studies are unavailable. The A345, S345, or F345 substitutions in the functional domain of D1 resulted in comparable hydrolysis rates following mutants of carboxyl-terminal processing protease (CtpA), while the G345 and V345 substitutions showed lower hydrolysis rates [77]. However, the P345 substitution resulted in complete inhibition [77]. 

ChlF has two unique S motifs in addition to the QD motif (Figure 3) that are likely hydrolysis sites with potentially lower hydrolysis rates. The H-bond between Y_Z_ and H190 of ChlF is potentially retained, although there may be H-bonds with other amino acids when the Mn_4_CaO_5_ cluster is depleted or uncoupled. Hence, electron transfer from Y_Z_ to P_680_^+^ is possible without the coupled functional Mn_4_CaO_5_ cluster, although it could be slower [2,78,79]. Therefore, a long-lived P_680_^+^ may exist in the PSII-ChlF complex that functions as a powerful oxidant with a redox potential of approximately 1.13 V [78].

Replacement of an MG motif in D1 with a unique QD motif in ChlF facilitates the production of Chl *f* in vivo. This suggests that the QD motif is the most probable active site of the enzyme, although it is unknown whether the close proximity of D128 is critical [19]. Further work is required to determine whether photoinhibition (or free radical formation) in the PSII-ChlF complex in fact catalyzes Chl *f* production.

## Data Availability

Not applicable.

## Supplementary Materials

The following supporting information can be downloaded at: https://www.mdpi.com/article/10.3390/microorganisms11092305/s1, Figure S1: Detailed H-bonds surrounding cofactor Pheo *a* in the models; Figure S2: Simulated models of ChlF containing chlorophyll *f* at different rotamers of protonated D128; Table S1: Genetic information of selected 19 cyanobacteria; Table S2: Modeling results of ChlF synthase, FRL-D1, WL-D1, FRL-CP43L, and WL-CP43 of *H. hongdechloris* against template PDB3WU2 with or without the inclusion of H atoms assigned during modeling; Table S3: H-bonding distances (Å) between pheophytin 13^1^-keto and 13^2^-keto groups and D1 of *T. vulcanus* (PDB3WU2), WL-D1, FRL-D1, and ChlF synthase from *H. hongdechloris* by the amino acids Q130/E130, Y126, and Y147 or F147, respectively. References [80,81,82,83] are cited in the supplementary materials.
