# Supplementary material for: Modeling the Characteristic Residues of Chlorophyll f Synthase (ChlF) from Halomicronema hongdechloris to Determine Its Reaction Mechanism
Source: Microorganisms. 2023 Sep 13;11(9):2305. doi: 10.3390/microorganisms11092305 (PMC10535343; doi:10.3390/microorganisms11092305)
Supplement: Supplementary file 1 [file microorganisms-11-02305-s001.zip › microorganisms-2608506-Supplementary Material.pdf]

Table S2. Modelling results of ChlF synthase, FRL-D1, WL-D1, FRL-CP43L, and WL-CP43 of *H. hongdechloris* against template PDB3WU2 with or without the inclusion of H atoms included during modelling.

Proteins	Modelling Trials (Chimera)	GA341[a,1] without / with H-atoms	zDOPE[b,2] without / with H-atoms	Estimated RMSD[c,3] without / with H-atoms	Estimated overlap (3.5 Å)[d,3] without / with H-atoms
ChlF synthase Full sequence	1	1.00 / 1.00	1.52 / 1.47	23.776 / 25.548	0.060 / 0.080
	2	1.00 / 1.00	1.58 / 1.46	24.042 / 23.954	0.070 / 0.060
	3	1.00 / 1.00	1.52 / 1.44	23.779 / 23.799	0.063 / 0.057
	4	1.00 / 1.00	1.56 / 1.53	24.09424.480	0.092 / 0.055
	5	1.00 / 1.00	1.61 / 1.49	25.223 / 25.285	0.131 / 0.065
FRL-D1 Full sequence	1	1.00 / 1.00	1.20 / 1.03	20.639 / 16.456	0.113 / 0.174
	2	1.00 / 1.00	1.20 / 1.13	20.928 / 21.932	0.074 / 0.079
	3	1.00 / 1.00	1.10 / 1.13	16.198 / 19.448	0.221 / 0.161
	4	1.00 / 1.00	1.15 / 1.12	21.084 / 19.297	0.104 / 0.109
	5	1.00 / 1.00	1.12 / 0.99	18.465 / 16.865	0.221 / 0.206
FRL-CP43 Full sequence	1	1.00 / 1.00	0.93 / 0.84	10.762 / 8.490	0.621 / 0.656
	2	1.00 / 1.00	0.89 / 0.85	15.052 / 7.978	0.316 / 0.713
	3	1.00 / 1.00	0.94 / 0.86	15.378 / 8.274	0.384 / 0.710
	4	1.00 / 1.00	0.94 / 0.84	12.891 / 7.765	0.385 / 0.709
	5	1.00 / 1.00	0.99 / 0.91	17.472 / 12.162	0.191 / 0.522
Typical D1 (WL-D1) Full sequence	1	1.00 / 1.00	1.00 / 0.88	18.166 / 17.366	0.139 / 0.171
	2	1.00 / 1.00	1.08 / 1.01	17.920 / 19.036	0.148 / 0.108
	3	1.00 / 1.00	1.01 / 0.93	18.009 / 17.775	0.137 / 0.168
	4	1.00 / 1.00	0.98 / 0.92	15.388 / 17.055	0.200 / 0.175
	5	1.00 / 1.00	1.01 / 0.91	18.060 / 17.117	0.139 / 0.155
WL-CP43 Full sequence	1	1.00 / 1.00	0.93 / 0.86	1.180 / 1.378	0.934 / 0.934
	2	1.00 / 1.00	0.94 / 0.80	1.042 / 1.312	0.988 / 0.934
	3	1.00 / 1.00	0.85 / 0.84	1.054 / 1.400	0.988 / 0.922
	4	1.00 / 1.00	0.88 / 0.81	1.316 / 1.403	0.928 / 0.918
	5	1.00 / 1.00	0.94 / 0.87	1.033 / 1.383	0.988 / 0.935
ChlF synthase N-ter Δ35/C-ter Δ20	1	- / 1.00	- / 0.96	- / 2.756	- / 0.895
	2	- / 1.00	- / 0.94	- / 2.825	- / 0.978
	3	- / 1.00	- / 0.90	- / 2.814	- / 0.994
	4	- / 1.00	- / 1.02	- / 3.019	- / 0.931
	5	- / 1.00	- / 0.98	- / 2.596	- / 0.991
FRL-D1 N-ter Δ10/C-ter Δ16	1	- / 1.00	- / 0.87	- / 12.389	- / 0.707
	2	- / 1.00	- / 0.91	- / 15.618	- / 0.475
	3	- / 1.00	- / 0.91	- / 11.680	- / 0.697
	4	- / 1.00	- / 0.93	- / 15.833	- / 0.526
	5	- / 1.00	- / 0.80	- / 10.154	- / 0.649
Typical D1 (WL-D1) N-ter Δ10/C-ter Δ16	1	- / 1.00	- / 0.69	- / 8.564	- / 0.727
	2	- / 1.00	- / 0.76	- / 10.979	- / 0.713
	3	- / 1.00	- / 0.73	- / 8.668	- / 0.803
	4	- / 1.00	- / 0.71	- / 9.117	- / 0.745
	5	- / 1.00	- / 0.74	- / 11.168	- / 0.704
FRL-CP43 N-ter Δ6/C-ter Δ33	1	- / 1.00	- / 0.63	- / 6.341	- / 0.842
	2	- / 1.00	- / 0.63	- / 1.617	- / 0.872
	3	- / 1.00	- / 0.63	- / 5.275	- / 0.834
	4	- / 1.00	- / 0.62	- / 1.607	- / 0.872
	5	- / 1.00	- / 0.68	- / 5.018	- / 0.867
WL_CP43 N-ter Δ9	1	- / 1.00	- / 0.75	- / 1.423	- / 0.925
	2	- / 1.00	- / 0.69	- / 1.151	- / 0.978
	3	- / 1.00	- / 0.72	- / 1.405	- / 0.923
	4	- / 1.00	- / 0.70	- / 1.405	- / 0.931
	5	- / 1.00	- / 0.76	- / 1.391	- / 0.914
PDB3WU2-D1 Full sequence	1	- / 1.00	- / 1.12	- / 18.312	- / 0.198
	2	- / 1.00	- / 1.01	- / 15.713	- / 0.316
	3	- / 1.00	- / 1.03	- / 15.838	- / 0.397
	4	- / 1.00	- / 1.07	- / 15.813	- / 0.272
	5	- / 1.00	- / 1.14	- / 18.994	- / 0.128
PDB3WU2-D1 N-ter Δ10/C-ter Δ16	1	- / 1.00	- / 0.88	- / 2.677	- / 0.996
	2	- / 1.00	- / 0.86	- / 2.608	- / 0.964
	3	- / 1.00	- / 0.87	- / 2.613	- / 0.979
	4	- / 1.00	- / 0.88	- / 2.721	- / 0.963
	5	- / 1.00	- / 0.90	- / 7.316	- / 0.868

a, GA341 - model score derived from statistical potentials (1). A GA341 value > 0.7 generally indicates a reliable model, defined as ≥ 95% probability of correct fold.

b, zDOPE - normalized Discrete Optimized Protein Energy (DOPE), an atomic distance-dependent statistical score (2).

c, Estimated RMSD - TSVMod-predicted Cα root-mean-square deviation (RMSD) of the model from the native structure (3).

d, Estimated Overlap (3.5 Å) - TSVMod-predicted native overlap (3.5 Å), fraction of Cα atoms in the model within 3.5 Å of the corresponding atoms in the native structure after rigid-body superposition (3).

Full sequence represents the original sequence. 'N-ter Δ35/ C-ter trunc Δ20' refers to the truncated sequences by removing N-terminal and C-terminal amino acids which do not align with the crystal structure of PDB 3WU2. PDB3WU2 D1 sequence from *T. vulcanus* was used as a reference to determine accuracy of the modelling procedure.

References: 1, Melo et al., Protein Sci 11:430 (2002); 2, Shen and Sali, Protein Sci 15:2507 (2006); 3, Eramian et al., Protein Sci 17:1881 (2008)

Table S3. H-bonding distances (Å) between pheophytin 13^1-keto and 13^2-keto groups and D1 of *T. vulcanus* (PDB3WU2), WL-D1, FRL-D1, and ChlF synthase from *H. hongdechloris* by the amino acids Q130/E130, Y126, and Y147 or F147, respectively.

			Distances (Å		Angles (°)			
Protein	H-bond donor (**D**)	Acceptor (**A**)	D-A	H-A	D-A-A' (θ)	D-H-A	A'-A-D	A'-A-H
D1, PDB3WU2	Y126, **O**H	13^2-keto (**O**1D) PHO408.A	2.628	1.704	149	160	149	153
	Q130, **N**E2	13^1-keto (**O**BD) PHO408.A	2.868	1.951	129	150	129	125
	Y147, **O**H	17^3-keto (**O**1A) PHO408.A	2.807	1.993	152	141	152	145
	Q130, NH	Y126, O	3.046	2.121	147	152	147	140
D1 of *T. vulcanus* (PDB3WU2) model	Y126, **O**H	13^2-keto (**O**1D) PHO408.A	2.813	2.515	148	98	148	130
	Q130, **N**E2	13^1-keto (**O**BD) PHO408.A	3.305	2.319	159	169	159	163
	Y147, **O**H	17^3-keto (**O**1A) PHO408.A	2.907	2.420	150	111	150	137
	Q130, NH	Y126, O	2.993	2.137	151	143	151	142
WL-D1, model	Y126, **O**H	13^2-keto (**O**1D) PHO408.A	2.896	2.456	169	131	169	167
	Q130, **N**E2	13^1-keto (**O**BD) PHO408.A	3.640	3.120	142	114	142	144
	Y147, **O**H	17^3-keto (**O**1A) PHO408.A	2.917	2.692	138	94	138	121
	Q130, NH	Y126, O	3.079	2.251	150	140	150	140
FRL-D1, model	Y126, OH	13^2-keto (**O**1D) PHO408.A	3.175	2.588	165	120	165	156
	E130, OE2<br>E130, OE1	13^1-keto (**O**BD) PHO408.A	3.107<br>4.189	3.447<br>3.517	 135	62<br>129	149<br>135	136<br>125
	Y147, OH	17^3-keto (**O**1A) PHO408.A	2.627	2.081	142	114	142	130
	E130, NH	Y126, O	3.079	2.264	149	138	149	139
ChlF synthase, model	Y126, OH	13^2-keto (**O**1D) PHO408.A	3.078	2.281	164	133	164	163
	E130, OE2<br>E130, OE1	13^1-keto (**O**BD) PHO408.A	3.164<br>3.830	3.611<br>4.080	 126	55<br>68	136<br>126	147<br>135
	F147	17^3-keto (**O**1A) PHO408.A	[redacted]					
	E130, NH	Y126, O	3.051	2.203	150	142	150	141

To determine hydrogen bonds between pheophytin *a* and E130 of ChlF synthase and FRL-D1 from H. hongdechloris, and, H-atoms were manually added and the protonation states of E130 were compared (OE1 and OE2) individually. The OE2 position of E130 was preferred. Red fonts indicate the manually measured potential H-bond distances, which were not detected in the original H-bond analysis.

This implies that direct H-bonds are not typically possible between E130 and ketone of pheo *a* since both are electron acceptors and there needs to be an electron donor e.g. O-H, N-H group. At physiological pH Glu has C=O and C-O (fully deprotonated), however, E130 strengthens H-bonds due to presumed protonatation of the R-group of E130 at physiological pH (Shibuya *et al.* 2010). Uniprot P51765 (PsbA) was used for modeling as the D1 subunit from PDB 3WU2.

Shibuya, Y., Takahashi, R., Okubo, T., Suzuki, H., Sugiura, M., and Noguchi, T. (2010) Hydrogen Bond Interactions of the Pheophytin Electron Acceptor and Its Radical Anion in Photosystem II As Revealed by Fourier Transform Infrared Difference Spectroscopy. *Biochemistry* **49**, 493-501

Supporting Figures

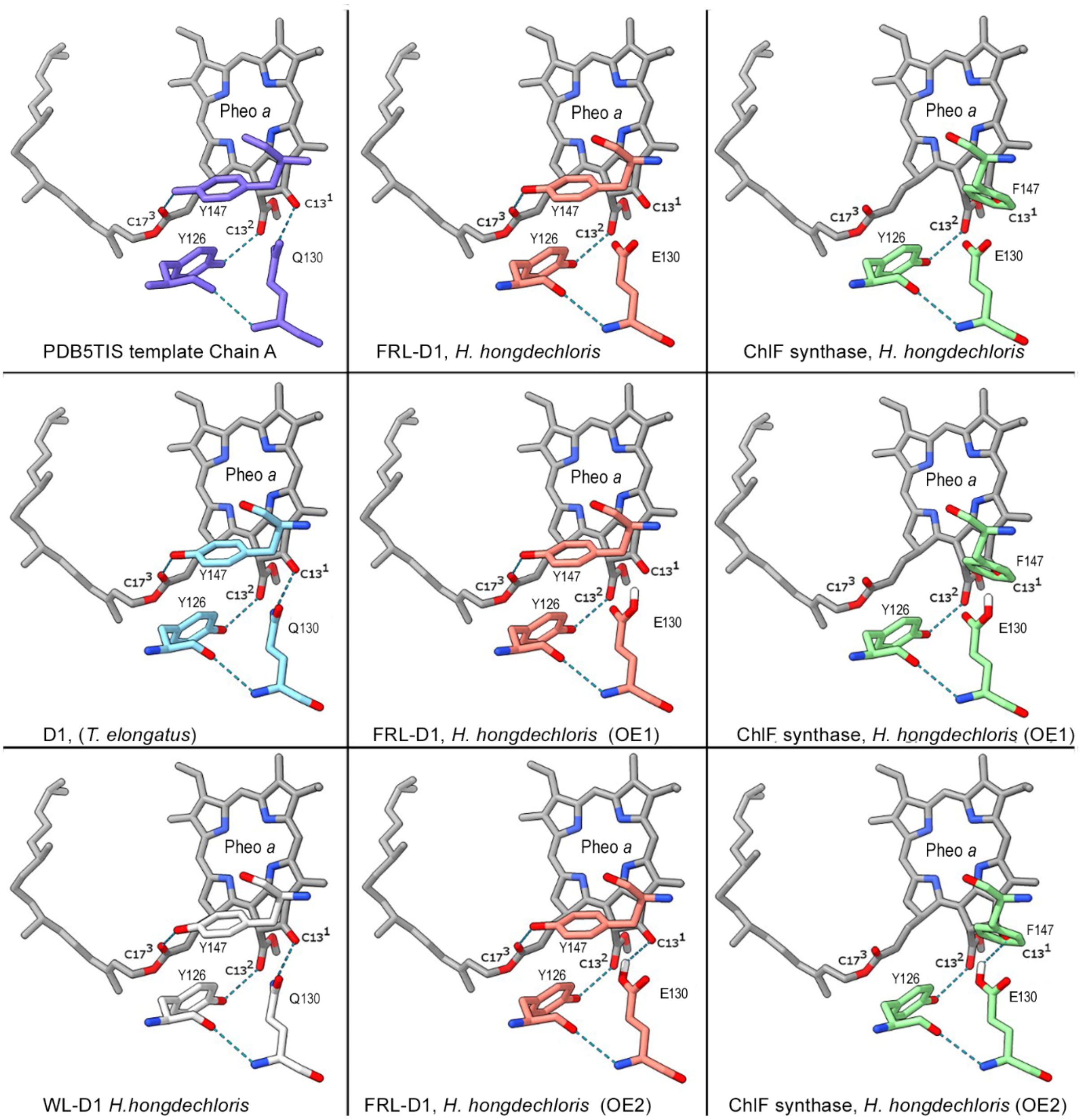

Figure S1. Detailed H-bonds surrounding cofactor Pheo *a* in the models
Modelling of WL-D1, FRL-D1, and ChlF from *H. hongdechloris* was performed using Swiss-model, with E130 protonated at OE2 using Chimera-X. The conserved H-bonds of a WL-D1 subunit (with very similar distances) is shown as a positive control to indicate the validity of the modelling. The Y147F substitution in ChlF indicated that no H-bond was detected at C17^3-Pheo *a*. Chlorophyll bonds are in grey; residues from the *T. elongatus* D1 template are in light blue; residues from *H. hongdechloris* WL-D1 are in white; residues from *H. hongdechloris* FRL-D1 are in orange; and residues from *H. hongdechloris* ChlF are in green; dark blue rods, nitrogen atoms; red rods, oxygen atoms; white rods, protonated sites.

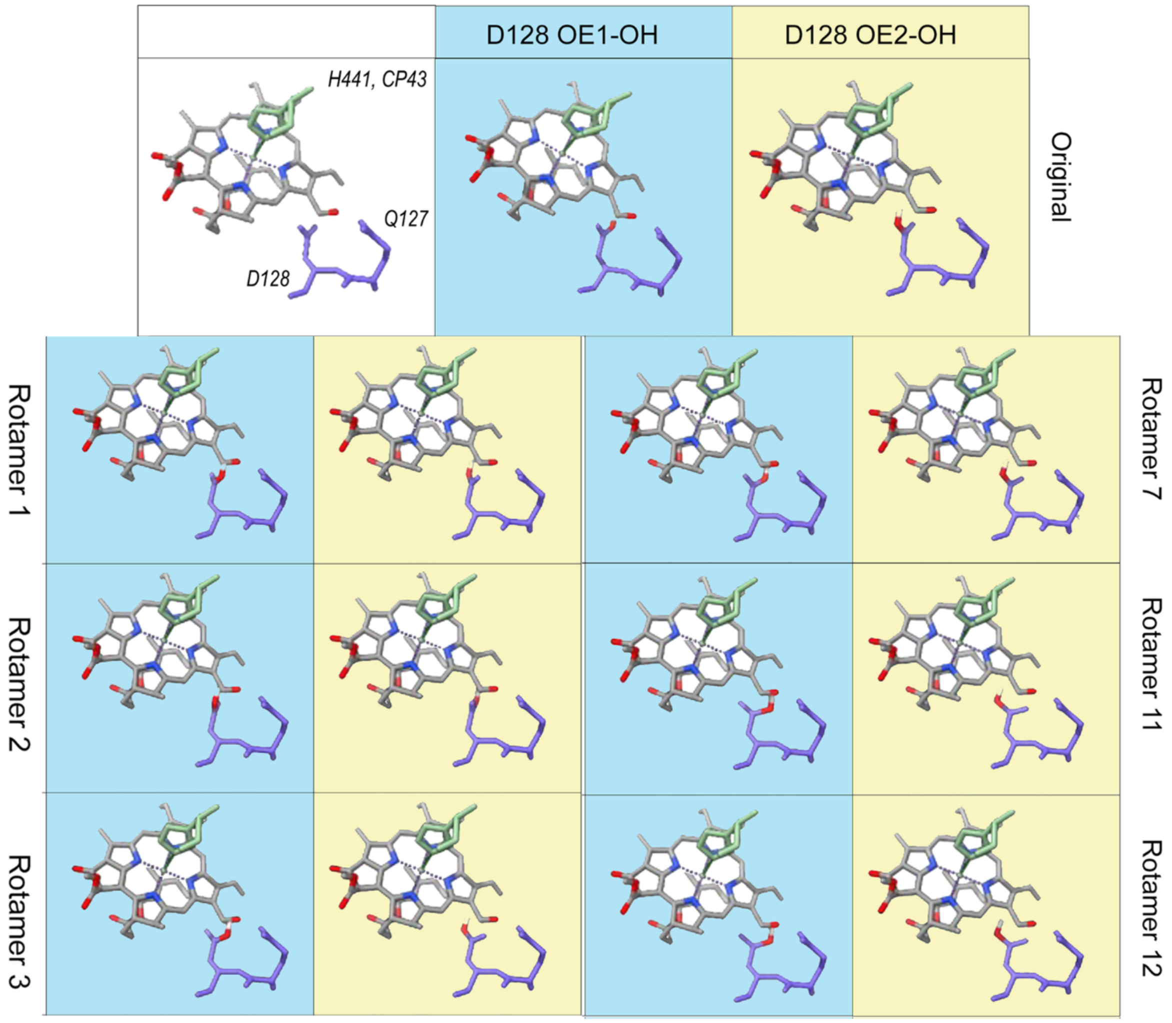

Figure S2. Simulated models of ChlF containing chlorophyll *f* at different rotamers of protonated D128.

A. non-protonated D128 in Chl *f*-interacting ChlF; B, protonated D128 at OE1 (blue shaded); C, protonated D128 at OE2 (yellow shaded). Modelling of PSIIFRL (ChlF) with Chl *f* (nominal position a) and rotamers of Asp128 without amino acid clashes. Rotamers with amino acid clashes were not considered. No H-bonds were detected after H-bond analysis of the selected rotamer. Chlorophyll molecular bonds are in grey; residues 127 and 128 of ChlF are in purple-blue. The histidine (H441) of CP43 is in green; dark blue rods represent nitrogen atoms; red rods are oxygen atoms; dotted blue lines are H-bonds.
